# Tocotrienols Reverse Cardiovascular, Metabolic and Liver Changes in High Carbohydrate, High Fat Diet-Fed Rats

**DOI:** 10.3390/nu4101527

**Published:** 2012-10-22

**Authors:** Weng-Yew Wong, Hemant Poudyal, Leigh C. Ward, Lindsay Brown

**Affiliations:** 1 School of Biomedical Sciences, The University of Queensland, Brisbane 4072, Australia; Email: j.wong4@uq.edu.au (W.-Y.W.); h.poudyal@uq.edu.au (H.P.); 2 School of Chemistry and Molecular Bioscience, The University of Queensland, Brisbane 4072, Australia; Email: l.ward@uq.edu.au; 3 Department of Biological and Physical Sciences, The University of Southern Queensland, Toowoomba 4350, Australia

**Keywords:** tocotrienols, obesity, cardiovascular, liver

## Abstract

Tocotrienols have been reported to improve lipid profiles, reduce atherosclerotic lesions, decrease blood glucose and glycated haemoglobin concentrations, normalise blood pressure *in vivo* and inhibit adipogenesis *in vitro*, yet their role in the metabolic syndrome has not been investigated. In this study, we investigated the effects of palm tocotrienol-rich fraction (TRF) on high carbohydrate, high fat diet-induced metabolic, cardiovascular and liver dysfunction in rats. Rats fed a high carbohydrate, high fat diet for 16 weeks developed abdominal obesity, hypertension, impaired glucose and insulin tolerance with increased ventricular stiffness, lower systolic function and reduced liver function. TRF treatment improved ventricular function, attenuated cardiac stiffness and hypertension, and improved glucose and insulin tolerance, with reduced left ventricular collagen deposition and inflammatory cell infiltration. TRF improved liver structure and function with reduced plasma liver enzymes, inflammatory cell infiltration, fat vacuoles and balloon hepatocytes. TRF reduced plasma free fatty acid and triglyceride concentrations but only omental fat deposition was decreased in the abdomen. These results suggest that tocotrienols protect the heart and liver, and improve plasma glucose and lipid profiles with minimal changes in abdominal obesity in this model of human metabolic syndrome.

## 1. Introduction

Dietary changes have been perceived as the first-line intervention in metabolic syndrome, targeting insulin sensitivity and preventing or correcting the associated metabolic and cardiovascular abnormalities. Targeting illness with selected components of foods, defined as treatment with functional foods or nutrapharmacology, could provide protection against cardiovascular diseases and diabetes [1,2]. Functional or medicinal foods and phytonutrients are widely accepted for maintaining well-being, enhancing health, and modulating immune function to prevent specific diseases [3]. 

Vitamin E is a group of closely-related phytochemicals including the tocopherols and tocotrienols, with potential cardiovascular and metabolic health-promoting properties [4]. They share a common chromanol ring with the tocopherols having a saturated phytyl side chain, differing from the farnesyl side chain with three double bonds in the tocotrienols. Each group has α-, β-, γ- and δ-homologues [5].

While *in vitro* and *in vivo* studies on tocopherols demonstrated positive antioxidant and anti-atherogenic effects, the clinical evidence was inconclusive or even negative [6]. This lack of therapeutic value of the tocopherols makes it worthwhile to investigate the efficacy of the tocotrienols, as these homologues may have unique functions [7,8]. Tocotrienols have been claimed to possess neuroprotective, anticancer and cholesterol-lowering properties that are often not exhibited by tocopherols [7]. Neurodegeneration in mouse hippocampal HT4 neural cells was prevented with 250 nanomolar concentrations of α-tocotrienol, but not α-tocopherol [9]. This suggests that the molecular and therapeutic targets of the tocotrienols are distinct from those of the tocopherols [7]. In addition to their shared antioxidant activities, tocotrienols have anti-inflammatory [10] and anti-angiogenic activities unlike the tocopherols [11,12]. These observations are of particular note considering the low plasma tocotrienol concentrations achieved in such studies, suggesting powerful metabolic effects. These activities could play vital roles in attenuating metabolic syndrome. There is little data on the ability of tocotrienols to reverse chronic diet-induced changes in humans. Hence, this study has measured the changes following intervention with palm tocotrienol-rich fractions (TRF) in a rat model of chronic diet-induced cardiovascular, metabolic and liver changes [13,14]. Palm-derived TRF is as tocopherol-tocotrienol mixture, at a ratio of approximately 1:3. The mixture is a commercial product for human consumption from companies including Golden Hope Bioganic (Sime Darby), Carotech, Palm Nutraceuticals, Eisai and Davos Life Sciences. However, individual homologues have been difficult to obtain in sufficient quantities for chronic animal studies. 

## 2. Experimental Section

### 2.1. Rats and Diets

The experimental groups consisting of male Wistar rats (aged 9–10 weeks; weighing 329 ± 2 g, *n* = 32) were obtained from The University of Queensland Biological Resources unit and individually housed at the University of Southern Queensland’s Animal House Facility. All experimental protocols were approved by the Animal Experimentation Ethics Committee of the University of Southern Queensland, under the guidelines of the National Health and Medical Research Council of Australia. Rats were divided into 4 groups: (i) corn starch (C, *n* = 8), (ii) high carbohydrate, high fat (H, *n* = 8), (iii) C + tocotrienol-rich fraction (TRF) (CT, *n* = 8), (iv) H + TRF (HT, *n* = 8). This TRF contained α-tocotrienol (31.9%), β-tocotrienol (2.1%), γ-tocotrienol (24.8%) and δ-tocotrienol (18.3%) together with α-tocopherol (22.9%). 120 mg/kg/day palm TRF dissolved in vitamin E-stripped palm olein (240 mg TRF/mL palm olein, Malaysian Palm Oil Board, Malaysia) was given for the final 8 weeks of the 16 weeks protocol via once-daily oral gavage. This dose was chosen as the reported oral no-observed-adverse-effects level in male rats given a similar tocotrienol-tocopherol mixture [15]. All experimental groups were housed in a temperature-controlled, 12-h light/dark cycle environment with *ad libitum* access to water and food. Measurements of body weight and food and water intakes were taken daily to monitor the day-to-day health of the rats. Feed conversion efficiency (%) was calculated as: 


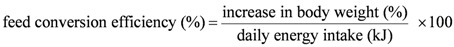


All group-specific diets were prepared in our laboratory. Corn starch diet was prepared by thorough mixing of corn starch, powdered rat feed (meat-free rat and mouse feed; Speciality Feeds, Glen Forrest, WA, Australia), Hubble, Mendel and Wakeman salt mixture (MP Biochemicals, Seven Hills, NSW, Australia) and water, while the corn starch and part of water were replaced with condensed milk, fructose and beef tallow in the high carbohydrate, high fat diet [14]. The drinking water in all high carbohydrate, high fat-fed rats was augmented with 25% fructose.

### 2.2. Echocardiography

Echocardiography was performed by trained cardiac sonographers at the Medical Engineering Research Facility, The Prince Charles Hospital, Brisbane, Australia. Rats were anaesthetised via intraperitoneal injection with Zoletil (tiletamine 15 mg/kg, zolazepam 15 mg/kg) and Ilium Xylazil (xylazine 10 mg/kg). Echocardiographic images were obtained using the Hewlett Packard Sonos 5500 (12 MHz frequency fetal transducer) at an image depth of 3 cm using two focal zones. Measurements of left ventricular posterior wall thickness and internal diameter were made using two-dimensional M-mode taken at mid-papillary level [13,16].

### 2.3. Body Composition Measurements

Dual-energy X-ray absorptiometric (DXA) measurements using a Norland XR36 DXA instrument (Norland Corp., Fort Atkinson, WI, USA) were performed on the rats after 16 weeks of feeding, 2 days before rats were killed for pathophysiological assessments. DXA scans were analysed using the manufacturer’s recommended software for use in laboratory animals (Small Subject Analysis Software, version 2.5.3/1.3.1; Norland Corp.) as previously described [17]. The precision error of lean mass for replicate measurements, with repositioning, was 3.2%. Visceral adiposity index (%) was calculated from wet weights of fat pads at euthanasia as:

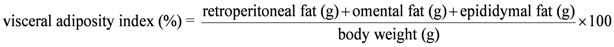

and expressed as adiposity per cent [18].

### 2.4. Assessment of Physiological Parameters

Systolic blood pressure was measured after 0, 4, 8, 12 and 16 weeks under light sedation with i.p. injection of Zoletil (tiletamine 15 mg/kg, zolazepam 15 mg/kg), using an MLT1010 Piezo-Electric Pulse Transducer (ADInstruments) and inflatable tail-cuff connected to a MLT844 Physiological Pressure Transducer (ADInstruments) and PowerLab data acquisition unit (ADInstruments, Sydney, Australia). Abdominal circumference was measured using a standard measuring tape under light sedation. Rats were killed with an intraperitoneal injection of pentobarbitone sodium (100 mg/kg). 

### 2.5. Oral Glucose Tolerance Test and Insulin Tolerance Test

Oral glucose tolerance tests were performed after 0, 8 and 16 weeks of diet. After 12 h of fasting, blood glucose concentrations were measured in blood samples taken from the tail vein. Subsequently, each rat was treated with glucose (2 g/kg) via oral gavage. Tail vein blood samples were taken every 30 min up to 120 min following glucose administration. The blood glucose concentrations were analysed with a Medisense Precision Q.I.D glucose meter (Abbott Laboratories, Bedford, MA, USA). 

For insulin tolerance testing, basal blood glucose concentrations were measured after 4–5 h of food deprivation as above. The rats were injected i.p. with 0.33 IU/kg insulin-R (Eli Lilly Australia, West Ryde, NSW, Australia), and tail vein blood samples were taken at 0, 30, 60, 90 and 120 min. Rats were withdrawn from the test if the blood glucose concentrations dropped below 1.1 mmol/L, and 4 g/kg glucose was administered immediately by oral gavage to reverse hypoglycaemia.

### 2.6. Organ Bath Studies

Changes in the responsiveness of thoracic aorta were defined using organ bath studies. Thoracic aortic rings (4 mm in length) were suspended in an organ bath chamber with a resting tension of 10 mN. Cumulative concentration-response (contraction) curves were measured for noradrenaline (Sigma-Aldrich Australia); concentration-response (relaxation) curves were measured for acetylcholine (Sigma-Aldrich Australia) or sodium nitroprusside (Sigma-Aldrich Australia) in the presence of a submaximal contraction to noradrenaline [14].

### 2.7. Isolated Heart Preparation

The left ventricular function of the rats in all treatment groups was assessed using the Langendorff heart preparation. Terminal anaesthesia was induced via i.p. injection of pentobarbitone sodium (100 mg/kg). Once anaesthesia was achieved, heparin (1000 IU) was injected into the right femoral vein. After removal of the heart, isovolumetric ventricular function was measured by inserting a latex balloon into the left ventricle connected to a Capto SP844 MLT844 physiological pressure transducer and Chart software on a Maclab system. All left ventricular end-diastolic pressure values were measured by pacing the heart at 250 beats per minute using an electrical stimulator. End-diastolic pressure was obtained starting from 0 mmHg up to 30 mmHg. The right and left ventricles were separated and weighed. Diastolic stiffness constant (κ, dimensionless) was calculated [16]. 

### 2.8. Organ Weights

Following euthanasia, the heart, liver, kidneys, visceral fat pads and spleen were removed and blotted dry for weighing. All organ weights were normalised relative to tibial length at the time of removal and presented in mg/mm.

### 2.9. Histology of Heart and Liver

Immediately after removal, heart and liver tissues were fixed in 10% buffered formalin with change of formalin every 3 days for 10 days to remove traces of blood from the tissue. The samples were then dehydrated and embedded in paraffin wax. Thin sections (5 μm) of left ventricle and the liver were cut and stained with haematoxylin and eosin stain for determination of inflammatory cell infiltration. Collagen distribution was observed in the left ventricle following picrosirius red staining. Laser confocal microscopy (Zeiss LSM 510 upright Confocal Microscope) was used to determine the extent of collagen deposition in selected regions.

### 2.10. Plasma Analyses

Blood was collected from the abdominal aorta following euthanasia and centrifuged at 5000× *g* for 15 min within 30 min of collection into heparinised tubes. Plasma was separated and transferred to Eppendorf tubes for storage at −20 °C before analysis. Plasma concentrations of total cholesterol, triglycerides, non-esterified fatty acids (NEFA), activities of plasma alanine transaminase (ALT) and aspartate transaminase (AST) were determined using kits and controls supplied by Olympus using an Olympus analyser (AU 400 Tokyo, Japan) [14].

### 2.11. Statistical Analysis

All data sets were represented as mean ± standard error of mean (SEM). Comparisons of findings between groups were made via statistical analysis of data sets using one-way and two-way analysis of variance (ANOVA). When interaction and/or the main effects were significant, means were compared using Newman-Keuls multiple-comparison *post hoc* test. A *p*-value of <0.05 was considered as statistically significant. All statistical analyses were performed using Graph Pad Prism version 5.00 for Windows.

## 3. Results

### 3.1. Cardiovascular Structure and Function

**Figure 1:**
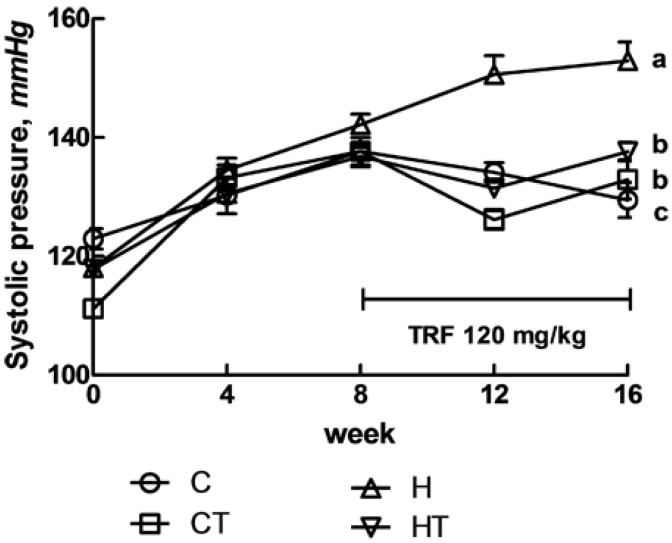
Tail-cuff measurement of systolic blood pressure recorded at 0, 4, 8, 12 and 16 weeks for C, CT, H and HT diet-fed rats. Data shown as means ± SEM. Endpoint means with different letters in each data set are significantly different. *n *= 8/group.

**Figure 2:**
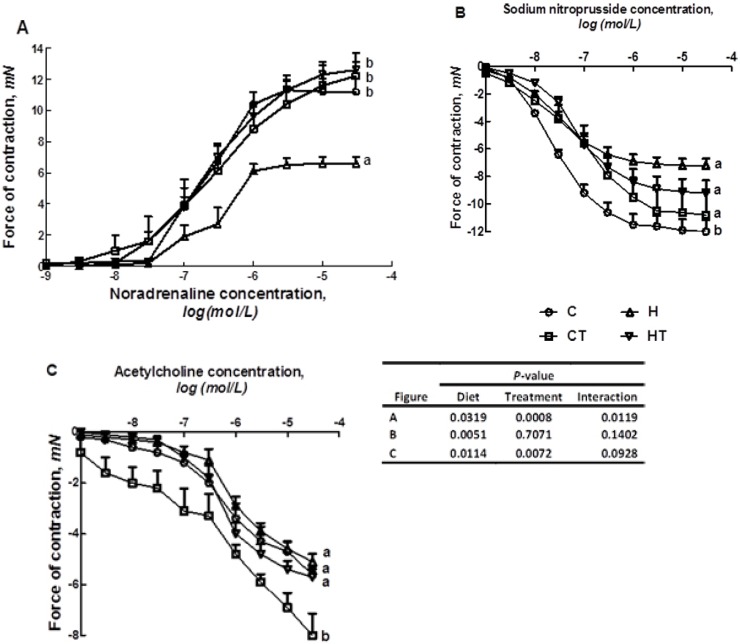
Cumulative concentration-response curves for noradrenaline (**A**), sodium nitroprusside (**B**) and acetylcholine (**C**) in thoracic aortic rings from C, CT, H and HT-diet fed rats. Data shown as means ± SEM. Endpoint means with different letters in each data set significantly differ. *n* = 8/group.

Feeding of the high carbohydrate, high fat (H) diet for 16 weeks increased systolic blood pressure compared with cornstarch (C) diet. With TRF supplementation for 8 weeks, blood pressure was normalised in rats fed with H diet (Figure 1). H feeding diminished noradrenaline contraction in isolated thoracic aortic rings (Figure 2A) and vascular relaxation responses to sodium nitroprusside (SNP) and acetylcholine (ACh) compared with C rats (Figure 2B,C). With TRF, thoracic aortic contractions to noradrenaline were improved (Figure 2A) but relaxation responses were unchanged (Figure 2B,C). 

**Figure 3:**
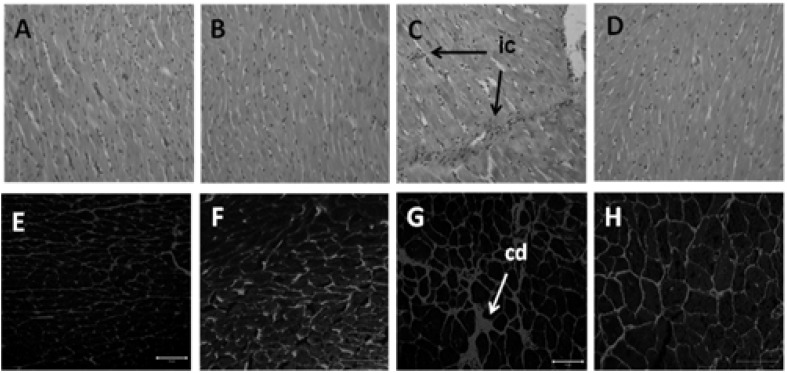
Haematoxylin and eosin staining of left ventricle (20×) showing inflammatory cells (marked as “ic”) as dark spots outside the myocytes in C (**A**), CT (**B**), H (**C**) and HT (**D**) diet-fed rats. Picrosirius red staining of left ventricular interstitial collagen deposition (40×) in C (**E**), CT (**F**), H (**G**) and HT (**H**) diet-fed rats; collagen deposition is marked as “cd”.

Compared to C group, H rats demonstrated eccentric hypertrophy, defined as an increased left ventricular weight and internal diameter in diastole (LVIDd) without any changes in relative wall thickness, with increased stroke volume and cardiac output (Table 1). H rats showed impaired systolic function seen as reduced fractional shortening. H rats showed decreased contractility, measured as maximal rate of positive rise of pressure (+dP/dt) and negative rise of pressure (−dP/dt), and LV developed pressure, in the isolated heart. Compared with H rats, relative wall thickness of HT rats was reduced. Systolic function was improved in TRF-treated rats, as shown by the increased fractional shortening, ejection fraction and cardiac output and decreased ejection time. Functionally, the increased diastolic stiffness in H rats was decreased in HT rats with improved −dP/dt. Histology of the H heart showed marked inflammatory cells infiltration (Figure 3C) and collagen deposition (Figure 3G) in the left ventricle compared with C rats. These changes were normalised with TRF treatment (Figure 3D,H).

**Table 1:** Changes in cardiovascular structure and function in C, CT, H and HT diet-fed groups.

Variables	C	CT	H	HT		*p*	
					Diet	Treatment	Interaction
LVIDd, *mm*	6.40 ± 0.21 ^a^	7.42 ± 0.22 ^b^	7.64 ± 0.26 ^b^	8.02 ± 0.10 ^b^	0.0001	0.0020	0.1280
LVPWd, *mm*	1.78 ± 0.03	1.86 ± 0.07	1.83 ± 0.03	1.85 ± 0.06	0.7014	0.2958	0.5658
Relative wall thickness	0.50 ± 0.02 ^a^^,^^b^	0.47 ± 0.04 ^a^^,^^b^	0.54 ± 0.02 ^a^	0.45 ± 0.01 ^b^	0.7876	0.0123	0.2189
Fractional shortening, *%*	48.0 ± 1.1 ^a^	51.5 ± 1.5 ^a^	42.5 ± 0.6 ^b^	48.6 ± 1.8 ^a^	0.0038	0.0011	0.3175
Ejection fraction, *%*	83.3 ± 0.8 ^b^^,^^c^	88.3 ± 1.1 ^a^	81.0 ± 0.6 ^c^	86.1 ± 1.5 ^a^^,^^b^	0.0414	<0.0001	0.9736
Heart rate, *bpm*	268 ± 22	288 ± 25	264 ± 24	308 ± 18	0.7448	0.1659	0.6057
Stroke volume, *mL*	0.28 ± 0.01 ^b^	0.38 ± 0.03 ^a^	0.42 ± 0.04 ^a^	0.46 ± 0.01 ^a^	0.0005	0.0096	0.3497
Cardiac output, *mL/min*	74.2 ± 3.9 ^a^^,^^b^	98.1 ± 7.3 ^a^^,^^b^	108.9 ± 12.25 ^b^	143.1 ± 9.1 ^c^	<0.0001	0.0045	0.091
LV developed pressure, *mmHg*	64.7 ± 8.8 ^a^	43.8 ± 4.5 ^b^	32.7 ± 2.3 ^b^	33.0 ± 2.6 ^b^	0.0003	0.0601	0.0540
(+)dP/dt, *mmHg/s*	1079 ± 104 ^a^	844 ± 89 ^a^^,^^b^	599 ± 39 ^b^	766 ± 97 ^a^^,^^b^	0.0031	0.6956	0.0266
(−)dP/dt, *mmHg/s*	614 ± 66 ^a^	507 ± 49 ^a^^,^^b^	359 ± 39 ^b^	512 ± 89 ^a^^,^^b^	0.0606	0.7235	0.0520
Diastolic stiffness, *κ*	22.8 ± 0.7 ^c^	23.5 ± 0.3 ^c^	28.8 ± 0.5 ^a^	26.4 ± 0.3 ^b^	<0.0001	0.1003	0.0037
Ascending aortic flow, *m/s*	0.90 ± 0.02	0.91 ± 0.07	0.95 ± 0.04	1.05 ± 0.07	0.1064	0.3305	0.4432
Descending aortic flow, *m/s*	0.87 ± 0.04	0.92 ± 0.09	0.93 ± 0.03	0.88 ± 0.07	0.8777	0.9607	0.4405
Ejection time, *s*	84.5 ± 3.2 ^a^	89.6 ± 3.0 ^a^	98.1 ± 2.5 ^b^	88.3 ± 2.2 ^a^	0.0349	0.3974	0.0112
Estimated LV mass, Litwin, *g*	0.89 ± 0.03	0.89 ± 0.07	0.98 ± 0.05	1.01 ± 0.03	0.0310	0.7471	0.7316
LV + septum wet weight, *mg/mm*	19.5 ± 0.7	17.8 ± 0.8	20.7 ± 0.9	20.3 ± 1.1	0.0427	0.2536	0.5082
Right ventricle wet weight, *mg/mm*	4.8 ± 0.7 ^a^^,^^b^	3.5 ± 0.4 ^b^	5.4 ± 0.5 ^a^	4.0 ± 0.4 ^a^^,^^b^	0.2726	0.0099	0.9280
Heart wet weight, *mg/mm*	24.8 ± 1.2	21.3 ± 0.8	25.3 ± 1.9	24.3 ± 1.1	0.2402	0.0789	0.4212
Systolic wall stress, *mmHg*	81.9 ± 1.8	80.6 ± 5.0	101.1 ± 8.1	96.1 ± 6.6	0.0060	0.5944	0.7547

Each value is mean ± SEM. Groups with letters different from others are significantly different (*p <* 0.05, *n* = 8).

### 3.2. Dietary Intake, Body Parameters and Lipid Profile

After 16 weeks, rats fed the H diet had increased body weight compared with C. H-fed rats had increased abdominal circumference and their visceral adiposity index was significantly higher than that of C rats (6.8% ± 0.6% compared to 3.6% ± 0.2%, respectively, Table 2). Consistent with the body weight results, fat mass and total visceral adipose tissue (retroperitoneal, epididymal and omental fat pads) in H rats were higher than in C rats. TRF treatment in H rats reduced omental, but not epididymal or perirenal fat pads, reduced abdominal circumference and increased food intake and lean mass. H rats had higher total cholesterol, triglyceride and NEFA plasma concentrations compared with C rats; TRF reduced triglyceride and NEFA but not total cholesterol concentrations (Table 2).

**Table 2:** Dietary intakes, body composition, metabolic indices and organ wet weights in C, CT, H and HT diet-fed rats.

Variables	C	CT	H	HT		*p*	
					Diet	Treatment	Interaction
Food intake, *g/day*	38.2 ± 1.0 ^a^	34.2 ± 0.8 ^b^	22.0 ± 0.9 ^c^	25.6 ± 0.5 ^d^	<0.0001	0.7595	0.0001
Water intake, *mL/day*	32.4 ± 2.8 ^a^	26.2 ± 1.0 ^b^	23.3 ± 1.2 ^b^	26.1 ± 1.4 ^b^	0.0132	0.3377	0.0148
Body weight gain, *%*	11.0 ± 1.0 ^a^^,^^b^	8.6 ± 0.9 ^b^	15.0 ± 1.5 ^a^	14.4 ± 1.4 ^a^	0.0005	0.2425	0.4535
Energy intake, *kJ/day*	443.2 ± 5.6 ^a^	397.5 ± 11.6 ^b^	480.1 ± 13.5 ^c^	556.3 ± 14.7 ^d^	<0.0001	0.2093	<0.0001
Feed conversion efficiency, *%*	2.4 ± 0.2	2.2 ± 0.3	3.0 ± 0.3	2.8 ± 0.2	0.0364	0.4079	0.9615
Bone mineral content, *g*	12.8 ± 0.1	13.0 ± 1.3	15.0 ± 0.6	14.9 ± 0.3	0.0092	0.9109	0.8408
Total fat mass, *g*	90.6 ± 7.0 ^b^	83.2 ± 6.2 ^b^	193.7 ± 20.1 ^a^	181.2 ± 9.8 ^a^	<0.0001	0.4197	0.8362
Total lean mass, *g*	335.9 ± 9.2 ^a^	288.8 ± 8.1 ^b^	257.7 ± 8.7 ^c^	270.1 ± 11.1 ^b^	<0.0001	0.0738	0.0036
Abdominal circumference, *cm*	19.8 ± 0.1 ^c^	19.7 ± 0.1^c^	22.4 ± 0.3 ^a^	21.8 ± 0.2 ^b^	<0.0001	0.0742	0.2337
Visceral adiposity index, *%*	3.6 ± 0.2 ^a^	4.3 ± 0.4 ^a^	6.8 ± 0.6 ^b^	6.6 ± 0.3 ^b^	<0.0001	0.6177	0.2546
*Tissue wet weight, mg/mm tibial length*							
Retroperitoneal fat	136.9 ± 11.6 ^a^	148.7 ± 11.0 ^a^	350.9 ± 29.3 ^b^	316.7 ± 21.9 ^b^	<0.0001	0.5777	0.2583
Epididymal fat	108.7 ± 8.8 ^a^	119.5 ± 12.9 ^a^	204.3 ± 23.3 ^b^	206.8 ± 16.7 ^b^	<0.0001	0.6883	0.8005
Omental fat	63.0 ± 6.7 ^c^	77.9 ± 9.2 ^c^	160.1 ±16.8 ^a^	127.8 ± 6.9 ^b^	<0.0001	0.4207	0.0365
Total abdominal fat	302.5 ± 23.5 ^a^	346.0 ± 29.1 ^a^	690.6 ± 71.2 ^b^	651.2 ± 35.9 ^b^	<0.0001	0.0018	0.0161
*Plasma lipid profile*							
Total cholesterol, *mmol/L*	1.23 ± 0.07 ^a^	1.63 ± 0.07 ^b^	1.74 ± 0.09 ^b^	1.60 ± 0.09 ^b^	0.0062	0.1252	0.0026
Triglyceride, *mmol/L*	0.34 ± 0.04 ^a^	0.44 ± 0.05 ^a^	0.90 ± 0.17 ^b^	0.60 ± 0.09 ^b^	0.0082	0.6539	0.2530
NEFA, *mmol/L*	0.98 ± 0.14 ^a^	1.46 ± 0.11^a^	2.28 ± 0.25 ^b^	1.74 ± 0.16 ^a^	0.0050	0.0394	0.0183
*Liver enzymes*							
ALT, *U/L*	24.8 ± 2.8 ^a^	28.7 ± 2.2 ^a^	43.9 ± 6.5 ^b^	25.8 ± 2.0 ^a^	0.0473	0.0779	0.0087
AST, *U/L*	67.1 ± 6.8 ^a^	65.7 ± 3.9 ^a^	96.6 ± 3.8 ^b^	58.0 ± 2.3 ^a^	0.0233	0.0002	0.0004
Liver wet weight, *mg/mm*	234.9 ± 10.6 ^a^	240.4 ± 8.8 ^a^	330.2 ± 13.8 ^b^	324.6 ± 4.6 ^b^	<0.0001	0.9966	0.5774
*Fasting plasma glucose, mmol* */* *L*							
0 week	3.5 ± 0.1	3.6 ± 0.1	3.7 ± 0.1	3.5 ± 0.1	0.0016	0.3305	0.0585
8 weeks	3.8 ± 0.2 ^a^	3.6 ± 0.2 ^a^	5.0 ± 0.1 ^b^	5.0 ± 01 ^b^	<0.0001	0.5322	0.5322
16 weeks	3.1 ± 0.2 ^c^	3.0 ± 0.1 ^c^	4.6 ± 0.2 ^a^	3.6 ± 0.2 ^b^	<0.0001	0.0050	0.0187

Each value is mean ± SEM. Groups with letters different from others are significantly different (*p < *0.05, *n* = 8).

### 3.3. Glucose Handling

**Figure 4:**
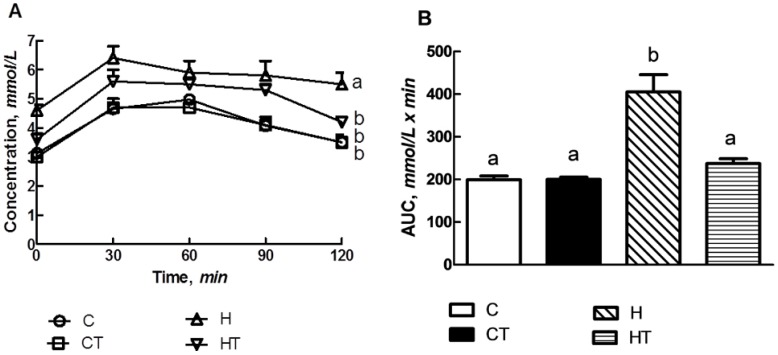
Oral glucose (2 g/kg) (**A**) and insulin (0.33 IU/kg) (**B**) tolerance in C, CT, H and HT-diet fed groups. Data shown as means ± SEM. Endpoint means with different letters in each data set significantly differ. *n* = 8/group.

H rats had higher fasting blood glucose concentration than C rats (Table 2) TRF treatment decreased the blood glucose concentrations in HT rats. The plasma glucose response to oral glucose loading was greater in H rats than C rats (Figure 4A). At 120 min, HT, CT and C rats had lower plasma glucose concentrations than H rats. In the insulin tolerance test, C, CT and HT rats had lower area under the curve than H rats (Figure 4B).

### 3.4. Hepatic Structure and Function

**Figure 5:**
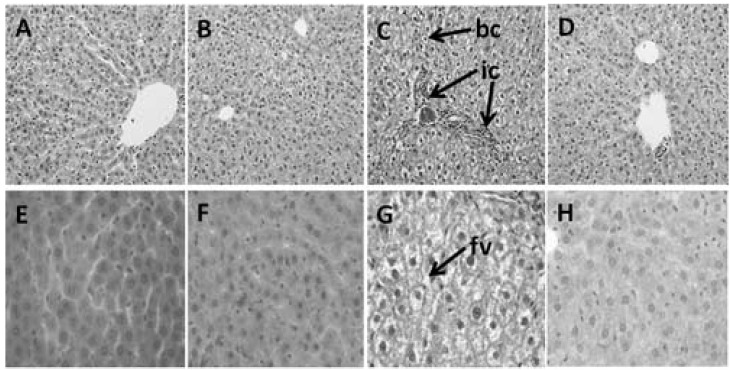
Haematoxylin and eosin staining of hepatocytes showing inflammatory cells around the portal region (marked as “ic”) (20×) in C (**A**), CT (**B**), H (**C**), and HT (**D**) diet-fed rats and hepatocytes with enlarged fat vacuole (marked as “fv”) (40×) and ballooned hepatocyte (marked as “bc”) in C (**E**), CT (**F**), H (**G**) and HT (**H**) diet-fed rats.

H rats had 1.8-fold greater plasma ALT and AST activities compared with C rats (Table 2). Liver weights of H rats were higher than C rats. With TRF, plasma AST and ALT activities were reduced by 41.2% and 40%, respectively, in HT rats. There were no differences in liver weight of HT and H rats. H rats developed fat vacuoles within the hepatocytes, showing ballooned hepatocytes with portal inflammatory cell infiltration compared with C rats (Figure 5E,G). Livers from the HT group showed a markedly attenuated degree of fatty change, with decreased fat vacuole size and number compared with the H group (Figure 5H). In addition, the HT rats displayed normalised portal inflammatory cells infiltration compared with H group (Figure 5C,D).

## 4. Discussion

This study shows improved metabolic parameters, and cardiovascular and liver structure and function, with smaller changes in abdominal obesity, in rats on a high carbohydrate, high fat diet administered TRF, a mixture of α-tocopherol, α-tocotrienol, γ-tocotrienol and δ-tocotrienol. We have shown that this high carbohydrate, high fat diet induces pathophysiological changes that resemble human metabolic syndrome [13], a syndrome that includes abdominal obesity, hyperglycaemia, dyslipidaemia, hypertension and fatty liver. Further, the changes in these rats can be reversed by interventions with compounds derived from foods, such as purple carrot extract high in anthocyanins [14], rutin from onions [19] and quercetin from apples [20]. Tocotrienols as important nutrients in food have been reported to change the individual parameters in the metabolic syndrome, including improved lipid profiles, decreased blood glucose concentrations and lowered blood pressures [4]. Oral supplementation of α-tocotrienol increased concentrations in many organs with skin, adipose tissue, liver, brain, ovaries and the heart as preferred destinations [21,22]. Treatment of the metabolic syndrome with tocotrienols could then improve the different aspects of metabolic syndrome including obesity, insulin resistance and cardiovascular disease, as an alternative to treating each risk factor with a separate intervention with the increased risks of polypharmacy [23]. TRF is Generally Recognised as Safe (GRAS) with no indication of significant adverse effects related to tocotrienol consumption at the dose used in this study; further, TRF products are commercially available with trade names including Tocomin, Gold TriE and Carotino TRF [24].

Several mechanisms have been proposed for the tocotrienol-induced improvements shown in this study. The reduced blood pressure with tocotrienols in Spontaneously Hypertensive Rats may result from increased total antioxidant status and superoxide dismutase activity with reduced lipid peroxidation [25] and increased production of prostacyclin [26]. Possible mechanisms for the decreased ventricular fibrosis include scavenging oxygen species to alleviate inflammation, and down-regulation of TGF-β1 which induces fibrosis [27]. Increases in collagen deposition are linked to the excess production of advanced glycation end-products (AGE) [28] that increase collagen cross-linking and deposition, increasing myocardial stiffness. TRF decreased AGE in STZ-induced diabetic rats [29]. Tocotrienols improved metabolic parameters including glucose utilisation and insulin sensitivity [29,30,31]. This may lead to decreased plasma triglycerides and non-esterified fatty acids since insulin is a potent suppressor of circulating NEFA concentrations through suppression of hormone-sensitive lipase, up-regulation of lipoprotein lipase [32] and maintaining a constant rate of free fatty acid re-esterification apart from enhancing glucose uptake and glycolysis, switching energy production from dominant fat oxidation to prevalent carbohydrate utilisation [33]. Tocotrienols reduce cancer growth by inhibiting angiogenesis [11,12]; δ-tocotrienol induced apoptosis in endothelial cells through growth factor-dependent phosphatidylinositol-3 kinase/PDK/Akt signalling [12]. Similar responses in adipose tissue should decrease growth of fat pads [4]. Further, our results indicate improved liver structure and function following TRF treatment.

A key change that may improve cellular survival is the reduced infiltration of inflammatory cells in the heart and liver, as shown in this study. Inflammation may initiate both insulin resistance and vascular dysfunction [34]. TRF inhibited the release of inflammatory mediators such as interleukin-6 and nitric oxide from macrophages *in vitro* [10]. While we have not measured changes in proinflammatory cytokines or other plasma biomarkers in this study, previous studies suggest that TRF inhibits production and release of TNF-α, TGF-β1 and IL-1β in STZ-induced diabetic rats [35]. The histology suggests that the anti-inflammatory responses are important and future studies on TRF and the individual homologues should include measurement of relevant biomarkers in plasma and their expression in tissues [36]. Damaged cells may show an increase in phosphorylated c-Src associated with pro-death signalling in myocytes [37] while increased Akt phosphorylation is generally associated with cardiomyocyte survival [38,39]. In neurones, α-tocotrienol blocked glutamate-induced death by suppressing glutamate-induced early activation of c-Src kinase [40], so tocotrienols may enhance cardiomyocte survival by preventing c-Src activation and enhancing Akt phosphorylation. 

TRF did not reduce visceral adiposity in this study, unlike other dietary interventions in this rat model [14,19,20,41]. In contrast, supplementation of rice bran tocotrienol mixture or α-tocopherol reduced body weight of F344 rats fed a high fat diet [42]. An *in vitro* study on 3T3-L1 cells suggested that α- and γ-tocotrienols reduced body fat by suppressing adipocyte differentiation and Akt phosphorylation [43]. Interpretation of the results is complicated by possible interactions between tocopherols and tocotrienols in TRF; as an example, tocopherol attenuated the cholesterol-lowering effect of γ-tocotrienol [44]. Tocopherols may decrease the responses to tocotrienols as the body prefers to absorb α-tocopherol rather than tocotrienols [5]. Further, preferential absorption has been reported for α-tocotrienol over γ-tocotrienol, δ-tocotrienol and α-tocopherol in thoracic duct-cannulated rats [45]. Differences in the number of methyl groups on the chromanol rings of the tocotrienols could affect the lipophilicity of the molecule and transportation to the lymphatic system via biological membranes [46]. In addition, interference by α-tocopherol has been recently reviewed [47]. α-Tocopherol may inhibit the uptake of α- and γ-tocotrienols to peripheral tissues such as heart, skin, aorta and perirenal adipose tissue, attributed to transport of tocotrienols and tocopherols to tissues by liver-dependent transport mechanisms [48].

## 5. Conclusions

TRF attenuated the structural and functional changes in the heart and liver associated with metabolic syndrome, and improved glucose metabolism and lipid profile. Prevention of inflammation may be the key mechanism. Although TRF showed minimal effects on obesity, studies with individual tocotrienols are warranted since the pure compounds may produce different responses to the mixture.
